# Riparian plant litter quality increases with latitude

**DOI:** 10.1038/s41598-017-10640-3

**Published:** 2017-09-05

**Authors:** Luz Boyero, Manuel A. S. Graça, Alan M. Tonin, Javier Pérez, Andrew J. Swafford, Verónica Ferreira, Andrea Landeira-Dabarca, Markos A. Alexandrou, Mark O. Gessner, Brendan G. McKie, Ricardo J. Albariño, Leon A. Barmuta, Marcos Callisto, Julián Chará, Eric Chauvet, Checo Colón-Gaud, David Dudgeon, Andrea C. Encalada, Ricardo Figueroa, Alexander S. Flecker, Tadeusz Fleituch, André Frainer, José F. Gonçalves Jr., Julie E. Helson, Tomoya Iwata, Jude Mathooko, Charles M’Erimba, Catherine M. Pringle, Alonso Ramírez, Christopher M. Swan, Catherine M. Yule, Richard G. Pearson

**Affiliations:** 1 Faculty of Science and Technology, University of the Basque Country (UPV/EHU), Leioa, 48940 Spain; 2 IKERBASQUE, Basque Foundation for Science, Bilbao, 48013 Spain; 3 College of Science and Engineering, James Cook University, Townsville, 4811 QLD Australia; 4 Doñana Biological Station (EBD-CSIC), Sevilla, 41092 Spain; 5 MARE-Marine and Environmental Sciences Centre, Department of Life Sciences, University of Coimbra, PT-3001-401 Coimbra, Portugal; 6 Laboratorio de Limnologia/AquaRiparia, Departamento de Ecologia, IB, Universidade de Brasília, 70910-900 Brasília, Federal District Brazil; 7 Department of Ecology, Evolution and Marine Biology, University of California, Santa Barbara, CA 93106 USA; 8 Department of Ecology and Animal Biology, University of Vigo, 36330 Vigo, Spain; 9 Wildlands Conservation Science, LLC, P.O. Box 1846, Lompoc, CA 93438 USA; 10 Department of Experimental Limnology, Leibniz Institute of Freshwater Ecology and Inland Fisheries (IGB), 16775 Stechlin, Germany; 11 Department of Ecology, Berlin Institute of Technology (TU Berlin), 10587 Berlin, Germany; 12 Department of Aquatic Sciences and Assessment, Swedish University of Agricultural Sciences, SE-75007 Uppsala, Sweden; 13 Laboratorio de Fotobiología, INIBIOMA, CONICET, Universidad Nacional Comahue, Quintral 1250, 8400 Bariloche, Argentina; 14 School of Biological Sciences, University of Tasmania, Private Bag 55, Hobart, Tasmania 7001 Australia; 15 Laboratorio de Ecologia de Bentos, Departamento Biologia Geral, ICB, Universidade Federal de Minas Gerais, 30161-970 Belo Horizonte, MG Brazil; 16 Centro para la Investigación en Sistemas Sostenibles de Producción Agropecuaria (CIPAV), Carrera 25 No. 6-62, Cali, Colombia; 17 EcoLab, Université de Toulouse, CNRS, INP, UPS, 118 Route de Narbonne, 31062 Toulouse, France; 18 Department of Biology, Georgia Southern University, Statesboro, Georgia 30458 USA; 19 School of Biological Sciences, The University of Hong Kong, Pokfulam, Hong Kong, SAR China; 20 Laboratorio de Ecología Acuática, Colegio de Ciencias Biológicas y Ambientales, Universidad San Francisco de Quito, Campus Cumbayá, P.O. Box 17, 1200 841 Quito, Ecuador; 21 Facultad de Ciencias Ambientales y Centro de Recursos Hidricos para la Agricultura y la Minería, Universidad de Concepción, Concepción, Chile; 22 Department of Ecology and Evolutionary Biology, Cornell University, Ithaca, NY 14853 USA; 23 Institute of Nature Conservation, Polish Academy of Sciences, Mickiewicza 33, 31-120 Kraków, Poland; 24 Department of Ecology and Environmental Science, Umeå University, Umeå, Sweden; 25 Department of Arctic and Marine Biology, UiT The Arctic University of Norway, 9037 Tromsø, Norway; 26 Surface and Groundwater Ecology Research Group, Department of Biological Sciences, University of Toronto at Scarborough, 1265 Military Trail, Toronto, Ontario, M1C 1A4 Canada; 27 Department of Environmental Sciences, University of Yamanashi, Kofu, Yamanashi, 400-8510 Japan; 28 Department of Biological Sciences, Egerton University, PO Box 536, Egerton, Kenya; 29 Odum School of Ecology, University of Georgia, 30602 Athens, GA USA; 30 Department of Environmental Science, University of Puerto Rico, Río Piedras, San Juan, Puerto Rico, 00919 USA; 31 Department of Geography and Environmental Systems, University of Maryland, Baltimore County, Baltimore, MD 21250 USA; 32 School of Science, Monash University, Jalan Lagoon Selatan, Bandar Sunway, Selangor, 47500 Malaysia

## Abstract

Plant litter represents a major basal resource in streams, where its decomposition is partly regulated by litter traits. Litter-trait variation may determine the latitudinal gradient in decomposition in streams, which is mainly microbial in the tropics and detritivore-mediated at high latitudes. However, this hypothesis remains untested, as we lack information on large-scale trait variation for riparian litter. Variation cannot easily be inferred from existing leaf-trait databases, since nutrient resorption can cause traits of litter and green leaves to diverge. Here we present the first global-scale assessment of riparian litter quality by determining latitudinal variation (spanning 107°) in litter traits (nutrient concentrations; physical and chemical defences) of 151 species from 24 regions and their relationships with environmental factors and phylogeny. We hypothesized that litter quality would increase with latitude (despite variation within regions) and traits would be correlated to produce ‘syndromes’ resulting from phylogeny and environmental variation. We found lower litter quality and higher nitrogen:phosphorus ratios in the tropics. Traits were linked but showed no phylogenetic signal, suggesting that syndromes were environmentally determined. Poorer litter quality and greater phosphorus limitation towards the equator may restrict detritivore-mediated decomposition, contributing to the predominance of microbial decomposers in tropical streams.

## Introduction

About 90% of the plant material produced annually in terrestrial ecosystems escapes herbivory and enters the pool of dead organic matter^1, 2^. Some of this plant litter is stored in soils and sediments over long periods, but much of it is decomposed, often providing a key basal resource for food webs in both terrestrial and aquatic ecosystems^3–5^. Ultimately, the fate of this organic matter influences the global carbon cycle through the release or sequestration of carbon dioxide (CO_2_) and other greenhouse gasses^6^. Stream ecosystems contribute significantly to CO_2_ release^7^, with a substantial proportion of the emitted CO_2_ being derived from in-stream biological activity^8^. In these systems, plant litter comes from the surrounding riparian vegetation, and it is decomposed by invertebrate detritivores and microorganisms^9^. However, the relative role of these decomposers changes across large spatial scales, including latitudinal gradients. While litter-consuming detritivores play a fundamental role in streams at mid and high latitudes, decomposition near the equator is mainly due to microbes^10^. Understanding the factors driving this latitudinal gradient is important because changes in the relative role of microbial decomposers and detritivores lead to differences in the amount of CO_2_ produced in different regions of the planet, and understanding this spatial variation may help forecast future emissions^10^.

Large-scale patterns of detritivore abundance and diversity are probably important determinants of the latitudinal decomposition gradient: litter-consuming detritivores are scarcer and less diverse in many tropical areas^11^, possibly as a result of elevated temperatures that are unfavourable to detritivores (many of which are cool-adapted taxa)^12^ and the reduced dispersal abilities of tropical detritivores^13^. However, it has also been proposed that differences in decomposition rate across latitudes are influenced by changes in the characteristics of riparian plant litter^14^. There is much evidence that litter traits affect decomposition rates in streams: in particular, decomposition is reduced when concentrations of lignin^15, 16^ or tannins^17^ are high or when litter is particularly tough^18^ and is often reported to be enhanced when litter nutrient concentrations are high^19, 20^. Similar relationships have been found for litter decomposition in terrestrial ecosystems^21–24^.

It is unknown, however, whether riparian litter traits change systematically along latitudinal gradients, and comparative information for terrestrial plant litter is also scarce at the global scale. This contrasts with the large number of comparative studies on green leaves, which have been mostly motivated by an interest in plant-herbivore interactions, following Dobzhansky^25^. Green leaves are typically poorer in nutrients in the tropics than at higher latitudes^26, 27^, and possibly better defended against herbivory^1, 2^ (but see ref. 28). However, the very few studies that have explored litter traits globally have found that traits of litter can differ from those of green leaves^29^, partly because of differences in nutrient resorption efficiency across latitudes^30^. This highlights the importance of quantifying trait variation of litter, rather than assuming that patterns for green leaves also pertain to litter. The most comprehensive study of litter trait variation, which used a dataset of 638 plant species across 6 biomes, showed that litter from tropical forests had higher nitrogen (N) but lower phosphorus (P) than litter from other biomes^30^. The other two existing global studies examined a wider range of traits, but included a limited number of species. This includes a study of 16 plant species from 4 biomes reporting higher lignin and hemicellulose concentrations in tropical litter, higher N concentration in temperate litter, higher concentrations of phenols and tannins in Mediterranean litter, and higher concentrations of P and micronutrients such as magnesium (Mg) and calcium (Ca) in subarctic litter^23^. The other study, involving a total of 20 plant species from 5 biomes, found that tropical litter was tougher, had lower specific leaf area (SLA) and lower concentrations of Mg and Ca than litter from other biomes^19^.

Here we present the first comprehensive study assessing riparian litter quality at the global scale, encompassing 151 riparian plant species (Supplementary Table S1) from 24 sites on six continents, spanning 107° of latitude and a wide climatic gradient (Supplementary Table S2), and multiple litter traits relevant for decomposition. We explored latitudinal variation in the concentration of major nutrients (N, P and their ratio, and Mg), physical defences (SLA, used as an inverse proxy for toughness) and chemical defences (concentration of condensed tannins), and the influence of climatic factors and soil characteristics in determining patterns of variation. We also explored how traits might be linked in ‘trait syndromes’^31^ (for example, litter with high nutrient concentrations might also be associated with low concentrations of tannins and low toughness, resulting in overall high litter quality; or vice versa), and whether such syndromes might be determined by environmental drivers or species’ phylogenetic relatedness. We predicted that (i) litter trait variation would be closely related to gradients in precipitation and temperature (and hence latitude), with litter quality decreasing towards the equator, (ii) significant variation would also occur within climatic regions due to local climatic gradients (e.g., in relation to altitude and soil characteristics), and (iii) traits would be linked in high- or low-quality syndromes mostly determined by environmental drivers, but with an influence of phylogeny also apparent.

## Results

**Figure 1:**
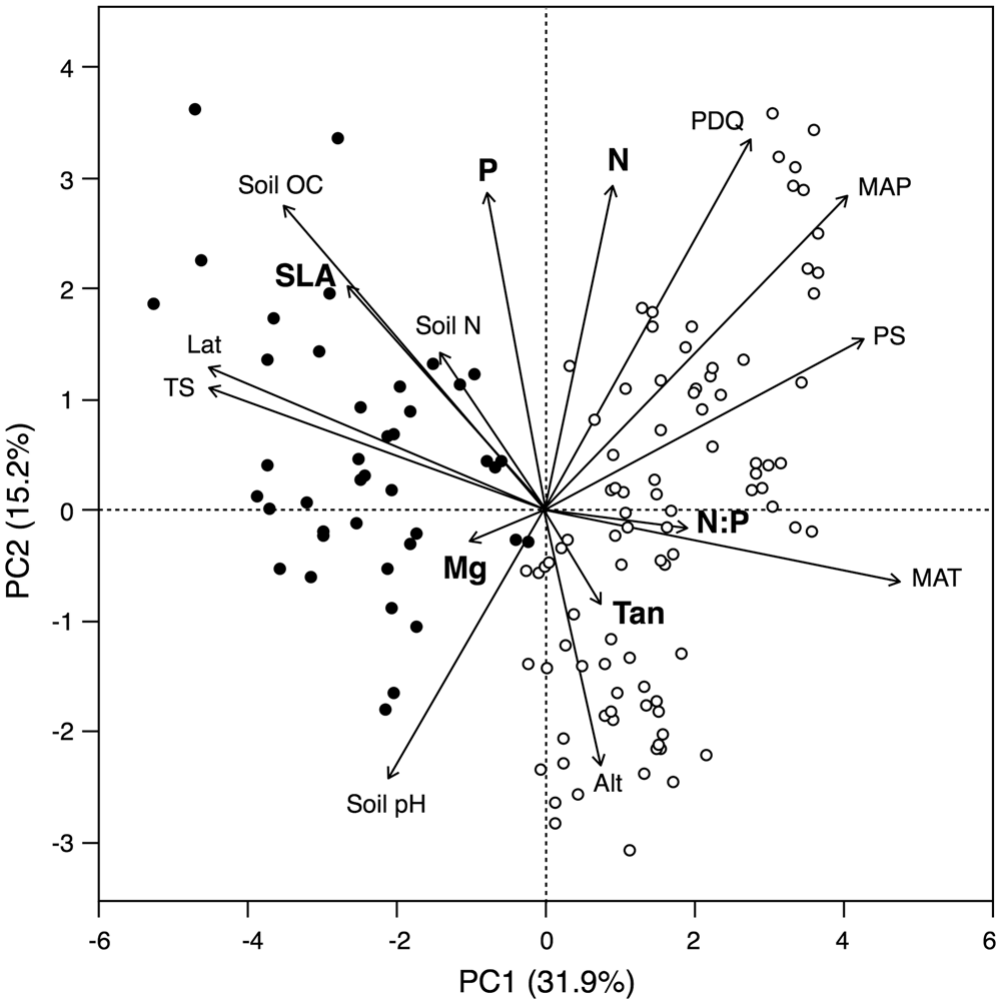
Principal component analysis (PCA) of litter traits [nitrogen (N) and phosphorus (P) concentration, N:P ratio, magnesium (Mg) and tannin (Tan) concentration, and specific leaf area (SLA); in bold letters] and environmental and spatial variables (mean annual temperature, MAT; mean annual precipitation, MAP; precipitation of the driest quarter, PDQ; temperature seasonality, TS; precipitation seasonality, PS; latitude, Lat; and altitude, Alt). Open and closed circles represent species from tropical and non-tropical regions (i.e., temperate, Mediterranean and boreal), respectively.

The two first axes of the Principal Component Analysis (PCA) explained 47.1% of the variance in litter traits and environmental variables (Fig. 1). The first axis (31.9%) was mostly related to latitude and temperature [both mean annual temperature (MAT) and temperature seasonality (TS)], with the tropical and non-tropical samples almost completely separated; the litter traits related to this axis were the N:P ratio (which increased with MAT and decreased with latitude and TS) and SLA, which showed the opposite pattern. The second axis (15.2%) was mostly related to altitude, precipitation of the driest quarter (PDQ), and two soil characteristics [pH and organic content (OC)]; the litter traits related to this axis were N and P concentrations and SLA (all inversely related to altitude and aridity). Tannins showed weak relationships with both axes, increasing towards lower latitudes and higher altitudes, and Mg showed a weak relationship with the first axis, increasing with latitude.

**Figure 2:**
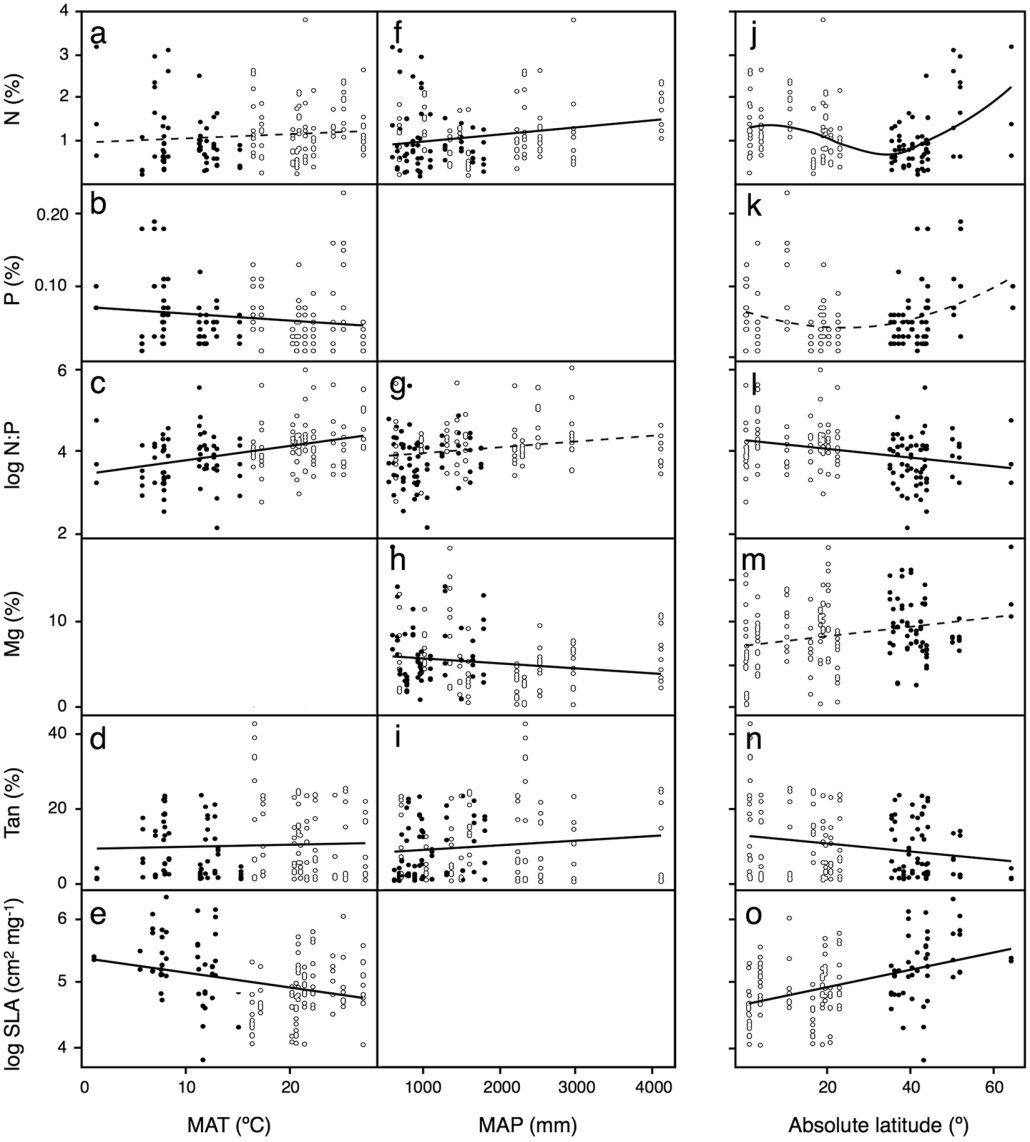
Variation of litter traits [nitrogen (N) and phosphorus (P) concentrations, log-transformed N:P ratio, magnesium (Mg) and tannin (Tan) concentrations, and log-transformed specific leaf area (SLA)] in relation to mean annual temperature (MAT), mean annual precipitation (MAP) and absolute degrees of latitude. Significant and non-significant relationships are depicted by solid and dotted lines, respectively. Fits for MAT **(a–e)** and MAP (**f**–**i**) derive from linear models that included multiple predictors; some graphs are omitted because MAT or MAP had been excluded from the final model; estimates and *p*-values are shown in Table 1. Fits for latitude (**j**–**o**) derive from additive models, which allowed analyses of non-linear relationships; *r* ^2^ and *p*-values are the following: N (*r* ^2^ = 0.18, *p* = 0.0024); P (*r* ^2^ = 0.06, *p* = 0.064); N:P (*r* ^2^ = 0.05, *p* = 0.0057); Mg (*r* ^2^ = 0.05, *p* = 0.212); Tan (*r* ^2^ = 0.04, *p* = 0.0156); SLA (*r* ^2^ = 0.32, *p* < 0.0001). Open and closed circles represent species from tropical and non-tropical regions, respectively.

Linear models explained 14–37% of the global variation in litter traits, and showed strong relationships between different traits (Table 1, Fig. 2). In particular, N and P concentrations were highly related, tannin concentration was tightly related to N and Mg concentrations, and a significant fraction of SLA variation was associated with tannin and P concentration. The most important environmental predictor for N concentration was mean annual precipitation (MAP; modulated by MAT), with some influence of soil pH; P concentration was associated with MAT, soil N concentration and soil pH; N:P was mostly related to MAT, modulated by MAP; Mg was correlated with soil N, MAP and soil pH; tannins were related to MAP, MAT and soil pH; and SLA was associated with MAT and soil N.

**Table 1:** Results of linear models examining global-scale variation of riparian litter traits [nitrogen (N) and phosphorus (P) concentrations, log-transformed N:P ratio, magnesium (Mg) and tannin (Tan) concentrations, and log-transformed specific leaf area (SLA)], depending on key climatic and soil predictors (mean annual temperature, MAT; mean annual precipitation, MAP; soil pH, SoilpH; and soil N concentration, SoilN) and on other litter traits.

Litter trait	Model	Factor	Estimate	Std. Error	*t*	*p*	%Variance
**N**	N ~ P + Tan + MAT + MAP + MAT × MAP + SoilpH						
	Variance explained: 37%	Intercept	1.0282	0.0383	26.87	<0.001	
	Total df: 164	**P**	**0**.**1880**	**0**.**0133**	**14**.**17**	<**0**.**001**	**66**.**30**
	Residual df: 157	**Tan**	**−0**.**0910**	**0**.**0232**	**−3**.**92**	<**0**.**001**	**15**.**34**
		MAT	0.0306	0.0442	0.69	0.489	3.05
		**MAP**	**0**.**4176**	**0**.**0652**	**6**.**41**	<**0**.**001**	**12**.**49**
		**MAT × MAP**	**−0**.**1550**	**0**.**0416**	**−3**.**72**	<**0**.**001**	**1**.**27**
		**SoilpH**	**0**.**1866**	**0**.**0340**	**5**.**49**	<**0**.**001**	**1**.**55**
**P**	P ~ N + MAT + SoilpH + SoilN						
	Variance explained: 34%	Intercept	0.0461	0.0014	33.92	<0.001	
	Total df: 164	**N**	**0**.**0143**	**0**.**0017**	**8**.**55**	<**0**.**001**	**84**.**88**
	Residual df: 159	**MAT**	**−0**.**0043**	**0**.**0022**	**−1**.**98**	**0**.**049**	**6**.**55**
		**SoilpH**	**0**.**0042**	**0**.**0019**	**2**.**24**	**0**.**026**	**3**.**33**
		**SoilN**	**0**.**0068**	**0**.**0019**	**3**.**65**	<**0**.**001**	**5**.**23**
**N:P**	Log N:P ~ Tan + MAT + MAP + MAT × MAP						
	Variance explained: 18%	Intercept	3.8256	0.0333	114.73	<0.001	
	Total df: 165	**Tan**	**−0**.**0753**	**0**.**0206**	**−3**.**66**	<**0**.**001**	**11**.**00**
	Residual df: 160	**MAT**	**0**.**2091**	**0**.**0515**	**4**.**06**	<**0**.**001**	**71**.**25**
		MAP	−0.0013	0.0487	−0.03	0.979	15.32
		**MAT × MAP**	**−0**.**0818**	**0**.**0412**	**−1**.**99**	**0**.**049**	**2**.**43**
**Mg**	Mg ~ N + P + N:P + MAP + SoilpH + SoilN						
	Variance explained: 14%	(Intercept)	4.3896	0.1723	25.48	<0.001	
	Total df: 164	**N**	**0**.**3710**	**0**.**1357**	**2**.**73**	**0**.**007**	**4**.**09**
	Residual df: 157	**P**	**−0**.**4528**	**0**.**2042**	**−2**.**22**	**0**.**028**	**3**.**51**
		**N:P**	**−0**.**8064**	**0**.**2391**	**−3**.**37**	**0**.**001**	**25**.**55**
		**MAP**	**−0**.**8824**	**0**.**2555**	**−3**.**45**	**0**.**001**	**21**.**49**
		**SoilpH**	**−0**.**9425**	**0**.**1800**	**−5**.**24**	<**0**.**001**	**6**.**95**
		**SoilN**	**−0**.**9481**	**0**.**1482**	**−6**.**40**	<**0**.**001**	**38**.**41**
**Tan**	Tan ~ N + Mg + MAT + MAP + MAT × MAP + SoilpH						
	Variance explained: 17%	Intercept	8.789	0.778	11.30	<0.001	
	Total df: 164	**N**	**−1**.**958**	**0**.**405**	**−4**.**83**	<**0**.**001**	**48**.**59**
	Residual df: 157	**Mg**	**−1**.**153**	**0**.**266**	**−4**.**34**	<**0**.**001**	**20**.**86**
		**MAT**	**−1**.**913**	**0**.**845**	**−2**.**26**	**0**.**025**	**1**.**64**
		**MAP**	**4**.**420**	**1**.**083**	**4**.**08**	<**0**.**001**	**12**.**06**
		**MAT × MAP**	**−1**.**714**	**0**.**741**	**−2**.**31**	**0**.**022**	**15**.**11**
		**SoilpH**	**−1**.**260**	**0**.**469**	**−2**.**69**	**0**.**008**	**1**.**74**
**SLA**	Log SLA ~ P + Tan + MAT + SoilN						
	Variance explained: 34%	Intercept	5.019	0.023	219.30	<0.001	
	Total df: 138	**P**	**0**.**106**	**0**.**024**	**4**.**36**	<**0**.**001**	**20**.**96**
	Residual df: 133	**Tan**	**−0**.**172**	**0**.**023**	**−7**.**53**	<**0**.**001**	**46**.**24**
		**MAT**	**−0**.**170**	**0**.**025**	**−6**.**94**	<**0**.**001**	**25**.**72**
		**SoilN**	**−0**.**136**	**0**.**031**	**−4**.**34**	<**0**.**001**	**7**.**08**

We show the proportion of variance explained by each model and the total and residual degrees of freedom (df; numerator df = 1 in all cases) and, for each factor, we show the mean estimate and standard error, *t*-statistic, *p*-value and proportion of variance explained; bold type indicates significant relationships at the *p* < 0.05 level.

Most traits showed significant latitudinal variation (Fig. 2): N concentration showed a significant (*p* = 0.002), nonlinear trend, being intermediate at low latitudes, decreasing at mid latitudes (≈20–40°) and increasing towards higher latitudes; an apparent curvilinear trend in P concentration was not significant (*p* = 0.064); the N:P ratio showed a significant linear trend (*p* = 0.006), decreasing with latitude; tannin concentration also decreased with latitude (*p* = 0.016); SLA strongly increased with latitude (*p* < 0.001); and Mg showed no trend (*p* = 0.21). None of the litter traits showed a phylogenetic signal, as indicated by non-significant tests for Pagel’s *λ* (N: *λ* = 0.28, *p* = 0.21; P: *λ* < 0.01, *p* = 1.00; N:P: *λ* < 0.01, *p* = 1.00; Mg: *λ* = 0.09, *p* = 0.38; tannins: *λ* = 0.13, *p* = 0.37; SLA: *λ* = 0.20, *p* = 0.63).

## Discussion

Our analyses revealed significant variation of key riparian litter traits across major climatic gradients, which were correlated with latitude and, to a lesser extent, altitude. Temperature appeared to be a major influence on the N:P ratio of litter, which showed a strong association with MAT and TS. The higher N:P ratios in warmer and seasonally less variable regions (i.e., towards the equator) agrees with findings for terrestrial plant litter in general^29^ and for green leaves^27^. These results provide support for the ‘soil substrate age’ hypothesis, which states that N and P concentrations of leaves are driven by the concentrations of these nutrients in soils, with tropical soils generally being more P-depleted than soils in temperate climates because of their often greater age (i.e. longer weathering) and higher leaching^27, 32^, which in the case of N is compensated for by N_2_ fixation^33, 34^. Consequently, tropical trees are expected to be more efficient at acquiring and resorbing P than N when compared to forest trees at higher latitudes^30^.

Although the relative concentrations of N and P were modulated by temperature and varied across latitudes, their absolute concentrations were more related to environmental gradients other than latitude (e.g., altitude and aridity), and to soil characteristics. The nonlinear variation of N concentration with latitude contrasts with reports of decreasing N concentrations with latitude^29^, but may be explained by the less humid nature of some of our study sites at ≈20° (e.g., the Brazilian ‘Cerrado’) and ≈40° (e.g., Spain). Other mid-latitude sites where litter had particularly low N concentration were in Argentina where litter was mostly from *Nothofagus* spp. that tend to have low N concentrations and high nutrient resorption during senescence^35^, and in Tasmania where litter was mostly from *Eucalyptus* spp. that, typically, are low in N^36^. In contrast, litter from Sweden, Poland and Germany had high N concentrations, and soils at these sites had relatively high organic carbon and/or N contents.

The concentration of P did not follow any latitudinal gradient, but decreased with MAT (possibly in relation to an altitudinal gradient, as suggested by the PCA) and was affected by soil characteristics. The seemingly contradictory inverse relationship of P with altitude may be because most of our high-altitude sites were in the tropics, whereas most lowland sites where P concentration was higher were in temperate areas (e.g., Canada, Germany, Maryland, Poland and Sweden), some of which also had soils with higher N concentration. Another study^29^ also found a decrease in P concentration with MAT for terrestrial litter in general, and no clear latitudinal gradient. Although Mg concentration in litter has been shown to increase from tropical to boreal sites^19^, our results did not confirm this trend, as the PCA only showed a weak association of Mg with latitude; Mg concentration was apparently greatest in litter at sites that were drier, and with soils that were richer in N (i.e., Argentina and Ecuador).

Traits related to plant defences against herbivory (i.e., tannin concentration and SLA) showed a latitudinal gradient, indicating the existence of tougher and less edible litter towards the equator. Tannin concentration decreased with latitude, and was affected both by MAT and MAP, but the interaction between these two factors made the interpretation difficult. Litter with the highest tannin concentration was from Ecuador, which showed moderately high values of MAP (≈2300 mm) and MAT (≈16 °C). SLA is an inverse proxy for litter toughness, so litter with higher SLA values is generally softer^37^. Like others, we found a strong latitudinal gradient for SLA^19^, indicating that litter is tougher towards the equator, with a major influence of MAT. Litter with the highest SLA (i.e., softer litter) was found in Poland, France and Maryland. However, this result contrasts with patterns for green leaves, where non-consistent patterns have been reported: a trend for greater leaf defences at higher latitudes^38^ or no latitudinal gradient^28^, and a preference of herbivores for leaves from higher latitudes^28, 39^. Differences between green leaves and litter are nevertheless expected because considerable changes in leaf chemistry occur during and after senescence^40–42^, including resorption prior to leaf shedding and leaching of soluble constituents after wetting of dead leaf tissue^30, 43^.

According to the ‘leaf economics spectrum’ hypothesis, leaf traits often co-vary, with little variation of trait relationships across climates^44^. In our study, N and P concentrations were indeed strongly correlated, and inversely related to tannin concentration. Moreover, there were positive relationships between N and Mg concentrations, and between P concentration and SLA. These relationships support the idea that leaves with high nutrient concentrations are less well defended, and are consistent with the existence of trait syndromes described for green leaves^31^. As we found no phylogenetic signal for the examined traits, such links among traits seem to be driven by environmental variation. However, this result should be viewed cautiously, as it contrasts with the significant phylogenetic signal in N and P concentrations and SLA reported in some regional studies using green leaves^45, 46^. These discrepancies are not surprising because the detection of phylogenetic signal can depend on the index and model used^47^ and the spatial scale investigated^48^. Thus, the amount of phylogenetic signal observed may vary under models of evolution not considered in this study. Moreover, the differences between our findings and those reported for green leaves could also be related to different nutrient resorption strategies in different species^30^, which could obscure patterns emerging for litter as opposed to green leaves.

Our study has demonstrated that the general tenet that litter quality varies with latitude holds true for riparian plants. This latitudinal gradient in litter quality is thus a plausible explanation for the observed gradient in litter decomposition in streams, in which litter-consuming detritivores only make a minor contribution in many tropical streams but play a key role at higher latitudes^10, 49^. Tropical riparian litter had more condensed tannins and was tougher, and both factors reduce litter consumption by detritivores^18, 50^. Tropical litter was not particularly poor in nutrients, but P was limiting compared to N^51^, which may cause greater stoichiometric imbalances in stream detritivores and, consequently, notable changes in litter decomposition rates^52^ and detritivore secondary production^53^. Thus, litter quality might affect decomposition not only directly by restricting detritivore feeding, but also indirectly through negative effects on populations of litter-consuming detritivores.

## Methods

### Field methods

**Figure 3:**
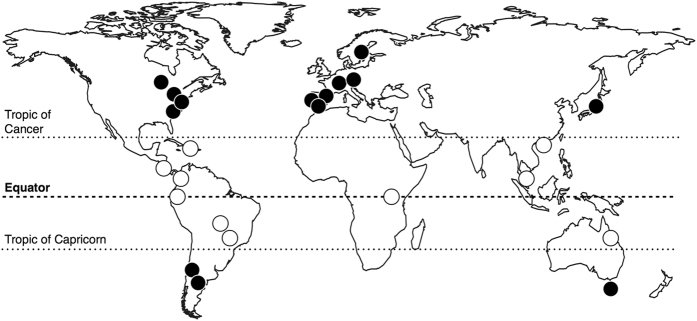
Location of 24 riparian litter collection sites; open and closed circles represent tropical and non-tropical regions, respectively. The map was created in the maps R package (Original S code by Richard A. Becker, Allan R. Wilks. R version by Ray Brownrigg. Enhancements by Thomas P Minka and Alex Deckmyn. (2016). maps: Draw Geographical Maps. R package version 3.1.1. https://CRAN.R-project.org/package=maps).

Leaf litter was collected from the riparian woody vegetation of 24 streams, located at latitudes between 64°N and 43°S (Fig. 3). All streams drained forested catchments with little human influence. The riparian vegetation was representative of the locality, with at least 70% canopy cover (Supplementary Table S2). We collected freshly fallen leaves from several species along each stream, to reflect the composition of leaves most commonly found in the streams. Leaves were collected from nets or from the forest floor just after abscission, so they had the same characteristics as leaves falling into the stream; additional selection criteria were no damage and no apparent signs of herbivory. The number of species collected varied from 3 to 7 at non-tropical sites (temperate, boreal and Mediterranean, all with species richness <20) and from 6 to 14 at tropical sites (where species richness was mostly >40); thus, the proportion of species collected was similar across sites (Supplementary Table S2). Litter was collected between 2011 and 2012 at times when most leaf fall occurs at each site (e.g., autumn at non-tropical sites and the dry season at some tropical sites); at sites where leaf fall proceeds slowly throughout the year, leaves were collected in nets that were checked periodically over longer periods. Leaves were air-dried to constant mass at room temperature and shipped to the University of Coimbra (Portugal), where all physicochemical analyses were performed.

### Litter traits

The litter traits considered were concentrations of N and P (% of dry mass) and their ratio, concentrations of Mg and condensed tannins (% of dry mass), and the SLA, which is the ratio of leaf area (cm) to dry mass (g). These traits cover three fundamental aspects of litter quality, namely nutrients and chemical and physical defences. Nutrient-rich litter is generally decomposed faster because it is preferred by microorganisms and detritivores^5^, and the N:P ratio is an indicator of which of the two nutrients is more likely to limit decomposition rates^54, 55^. Mg is an important component of invertebrate diets and its concentration in litter can affect decomposition^19, 23^. Condensed tannins are plant secondary compounds that can restrain microbial activity and detritivore feeding^5, 56^. Finally, the SLA is inversely related to leaf toughness and lignin concentration, suggesting that leaves with higher SLA decompose faster^37^.

We took ~1 g of air-dried leaves (n ≥ 4 leaves) of each species, removed their petioles, ground the leaves in a Retsch Mixer Mill MM 400 (Retsch GmbH, Haan, Germany), and dried the resulting powder at 45 °C for 48 h. To determine N concentration, we packed 0.5–0.7-mg portions into tin capsules and analysed them in an isotope-ratio mass spectrometer (IRMS Thermo Delta V advantage with a Flash EA, 1112 series; Thermo Fisher Scientific Inc., Waltham, MA, USA). Condensed tannins were measured using the acid butanol assay^57^ on 50-mg portions of leaf powder. Samples of 100 mg were combusted in a muffle furnace (550 °C, 8 h) and 5-mg portions of ash were dissolved in 25 mL of distilled water; a 5-mL aliquot of this solution was used for Mg determination by atomic absorption spectrometry (AAS, SOLAAR M Series equipment from Thermo–Unicam; Thermo Fisher Scientific Inc., Waltham, MA, USA). To determine P concentration, we acidified the remaining 20-mL aliquot with 1 mL of concentrated HCl, added deionized water for a final volume of 100 mL, and filtered the resulting solution through a Whatman GF/C filter (Whatman, Maidstone, UK). P was determined on the filtrate by the molybdate-blue method, and absorbance was measured at 880 nm on a Jenway 6715 UV/Vis spectrophotometer^58^. Additional leaves were rehydrated and ten 12-mm diameter discs were cut with a cork borer; the discs were then oven-dried at 45 °C for 48 h and weighed to determine SLA as the ratio of disc area (cm^2^) to leaf dry mass (mg).

### Environmental and spatial variables

We extracted several climatic variables from the WorldClim database version 1.3^59^ at the highest resolution (30 arc-seconds) using DIVA-GIS v7.5.0.0 (www.diva-gis.org) for each study site. These variables represented mean values and variability of temperature and precipitation for each site [mean annual temperature (MAT, °C), mean annual precipitation (MAP, mm), and temperature and precipitation seasonality (TS and PS, respectively, estimated as the standard deviation of monthly mean values × 100)] and an inverse proxy of aridity [precipitation of the driest quarter (PDQ, mm)]. We extracted the soil class^60^, soil pH, and soil organic carbon content (OC; g kg^−1^) for each study site from the Soil Grids database (www.soilgrids.org; resolution: 120 arc-seconds), and a value of soil N concentration (kg m^−2^ at 0 to 30 cm depth) was assigned to each study site depending on its soil class, based on Batjes^61^ (resolution: 30 arc-seconds). We recorded the absolute decimal latitude (degrees from equator) and altitude (m asl) of each study site on Google Earth.

### Data analyses

Firstly, we used Principal Component Analysis (PCA) to visualize the variation of litter traits across species in relation to the environmental and spatial variables using JMP 9.0.1 (www.jmp.com). All variables were converted to *z*-scores using the ‘scale’ function in the base package of R software^62^, to standardize units and obtain slopes comparable in magnitude^63^.

Secondly, we used linear models to examine the variation of each trait in relation to other traits (except for SLA, which had multiple missing values and was only used as a response variable) and the environmental and spatial predictors. Potential outliers in response variables were detected using Cleveland dotplots by sites (ggplot2 R package) and were removed (i.e., one observation each for N, P, Mg and SLA) (Supplementary Fig. S1). Two response variables (N:P ratio and SLA) presented multiple extreme observations for some sites (and thus a violation of the homogeneity assumption for linear models), so they were log-transformed to reduce the influence of extreme observations on the model fit^64, 65^. Before running the linear models we inspected bivariate scatterplots and Pearson correlations (‘chart.Correlation’ function in PerformanceAnalytics R package) to identify and remove any collinear environmental or litter trait predictor (r ≥ 0.60)^66^. As a result of this inspection we excluded latitude, altitude, TS, PS, PDQ and soil OC, which were strongly correlated with several other variables. Given that collinearity between N and P (r = 0.58) and MAT and MAP (r = 0.63) was near the threshold, we calculated variance inflation factors (VIFs) for all the predictors to ensure they would not inflate the variance of models. As all VIFs were below 4, they were maintained^66^ (Supplementary Table S3). All predictors were converted to *z*-scores. Different residual spread within sites was allowed through the use of a variance structure (‘VarIdent’ function in nlme package^67^; Zuur *et al*.^66^), the need for which was defined based on Akaike’s information criterion (AIC). Finally, we used a backward selection procedure based on AIC to define the best model for each litter trait (Supplementary Table S4). Briefly, we started with a model containing all individual predictors and the interaction between MAT and MAP (i.e., the full model), then sequentially removed the least significant predictor (to obtain a reduced or nested model), and tested model improvement based on AIC (using the ‘update’ and ‘anova’ functions to remove predictors and to compare full and reduced models, respectively) until we reached a model where all predictors were significant^65^. Models were fitted using the ‘gls’ (generalized least squares) function and restricted maximum likelihood (REML) method in the nlme R package. Regression plots were drawn with ggplot2 package^68^. The relative contribution of each predictor to the model was estimated using bootstrapping (‘lmg’ metric with ‘boot.relimp’ and ‘booteval.relimp’ functions of the relaimpo R package)^69^.

Thirdly, because we were interested in latitudinal gradients, and latitude was excluded from the linear models due to collinearity with MAT and MAP, we explored the latitudinal variation of litter traits using generalized additive mixed models (GAMM)^66, 70^. We used this type of model because, even if we expected linear relationships (i.e., lower litter quality towards the equator), initial data exploration showed non-linear patterns for some litter traits across the latitudinal gradient. Models were fitted with each litter trait as a response variable against absolute latitude fitted as a smoother, using a normal distribution and an identity link function (‘gamm’ function in the mgcv R package)^70^. Observations within each site (i.e., species data) were considered as a random component, and the variance structure was the same as described above for linear models. The need for both components was defined based on AIC.

Finally, we examined the phylogenetic signal of each litter trait – that is, the tendency for related species to share more similar trait values than species drawn at random from the phylogenetic tree^71^. For that purpose, we used a previously constructed, fossil-calibrated tree of angiosperms^72^ as a framework. We placed missing species in the tree next to nearest relatives using the tool ‘leafbud.py’ in Python 2.7 (Supplementary Methods), employing a method similar to Phylomatic^73^. Inserted species were assigned a branch length equal to their nearest relatives. Polytomies were randomly resolved using the tool ‘ete3’ in Python 2.7^74^. The new branch created by resolving the polytomy was assigned half of the length of the shortest child branch attached to the polytomous node. The child branches on the resolved nodes were adjusted to account for the added distance resulting in no change in the distance between any species and the root. We quantified the phylogenetic signal of litter traits using Pagel’s *λ* ^75^ in the R package phytools^76^; this index indicates stronger relationships between species traits and the phylogeny by the strength of its deviations from zero^77^. Under a Brownian motion model, where species inherit their traits from ancestors but then slowly diverge by small random steps occurring at a constant rate, Pagel’s *λ* is expected to be equal to 1, whereas values of 0 imply that there is no phylogenetic dependence^77^.

### Data availability

Data are available on the Open Science Framework online repository (https://osf.io/95cxb/?view_only=c1832aac5044452db4a8156d2caaeea5).

## Electronic supplementary material

Supplementary Information
