# Supplementary material for: Riparian plant litter quality increases with latitude
Source: Sci Rep. 2017 Sep 5;7:10562. doi: 10.1038/s41598-017-10640-3 (PMC5585321; doi:10.1038/s41598-017-10640-3)
Supplement: Supplementary file 1 — Supplementary Information [file 41598_2017_10640_MOESM1_ESM.pdf]

Riparian plant litter quality increases with latitude (Boyero et al.)

Supplementary Information

Table S1. List of riparian plant species analysed for each of 24 collection sites (see Table S2 for information on the sites).

Site	Family	Species
**Kenya**	Apocynaceae	*Tabernaemontana stapfiana*
	Euphorbiaceae	*Sapium ellypticum*
	Lamiaceae	*Vitex keniensis*
	Meliaceae	*Turraea abyssinica*
		Turraea holstii
	Moraceae	*Ficus natalensis*
		Ficus sycomorus
	Myrtaceae	*Syzygium guineense*
	Rutaceae	*Fagaropsis angolensis*
		Zanthoxylum gilletii
**Ecuador**	Actinidaceae	*Saurauia tomentosa*
	Asteraceae	*Critoniopsis occidentalis*
	Clusiaceae	*Clusia ducuoides*
	Euphorbiaceae	*Alchornea grandiflora*
	Fabaceae	*Erythrina edulis*
	Hypericaceae	*Vismia tomentosa*
	Lauraceae	*Ocotea insularis*
	Melastomataceae	*Tibouchina lepidota*
	Moraceae	*Ficus* aff. *guianensis*
		Ficus dulciaria
	Solanaceae	*Solanum* aff. *grandiflorum*
	Verbenaceae	*Citharexylum montanum*
**Colombia**	Euphorbiaceae	*Alchornea grandiflora*
	Malvaceae	*Heliocarpus americanus*
	Melastomataceae	*Meriania longifolia*
		Miconia caudata
	Moraceae	*Sorocea trophoides*
	Sapindaceae	*Cupania* cf. *latifolia*
**Malaysia**	Annonaceae	*Monocarpia marginalis*
	Burseraceae	*Canarium patentinervium*
	Dipterocarpaceae	*Dipterocarpus costulatus*
		Dipterocarpus elongatus
	Euphorbiaceae	*Cheilosa malayana*
		Macaranga tanarius
	Fabaceae	*Koompassia malaccensis*
	Malvaceae	*Neesia altissima*
	Moraceae	*Parartocarpus bracteatus*
	Myristicaceae	*Knema laurina*
	Poaceae	*Gigantochloa scortechinii*
**Costa Rica**	Combretaceae	*Terminalia oblonga*
	Fabaceae	*Lonchocarpus oliganthus*
		Zygia longifolia
	Malvaceae	*Luehea seemannii*
	Moraceae	*Castilla elastica*
		Ficus insipida
		Ficus tonduzii
	Myristicaceae	*Virola koschnyi*
	Rubiaceae	*Simira maxonii*

	Urticaceae	*Cecropia obtusifolia*
**Brazil (Brasilia)**	Apocynaceae	*Aspidosperma discolor*
	Burseraceae	*Protium heptaphyllum*
		Protium spruceanum
	Chrysobalanaceae	*Licania kunthiana*
	Fabaceae	*Pterodon pulenscens*
		Tachigali subvelutina
	Icacinaceae	*Emmotum nitens*
	Lauraceae	*Ocotea corymbosa*
	Malpighiaceae	*Heteropterys anoptera*
	Phyllanthaceae	*Richeria grandis*
**Puerto Rico**	Araliaceae	*Dendropanax arboreus*
	Arecaceae	*Prestoea acuminata*
	Burseraceae	*Dacryodes excelsa*
	Elaeocarpaceae	*Sloanea berteriana*
	Euphorbiaceae	*Alchornea latifolia*
	Fabaceae	*Ormosia krugii*
	Lauraceae	*Ocotea leucoxylon*
	Meliaceae	*Guarea guidonia*
		Swietenia macrophylla
	Sapotaceae	*Manilkara bidentata*
	Urticaceae	*Cecropia schreberiana*
**Australia (Queensland)**	Celastraceae	*Perrottetia arborescens*
	Commelinaceae	*Pollia macrophylla*
	Icacinaceae	*Apodytes brachystylis*
	Monimiaceae	*Wilkiea pubescens*
	Myrsinaceae	*Ardisia brevipedata*
	Myrtaceae	*Syzygium johnsonii*
	Oleaceae	*Chionanthus axillaris*
	Pandanaceae	*Freycinetia excelsa*
	Proteaceae	*Helicia nortoniana*
	Symplocaceae	*Symplocos cochinchinensis* var. *gittonsii*
**Brazil (Belo Horizonte)**	Burseraceae	*Protium brasiliense*
		Protium heptaphyllum
	Euphorbiaceae	*Sebastiania brasiliensis*
	Melastomataceae	*Leandra scabra*
		Miconia chartacea
	Meliaceae	*Cabralea canjerana*
	Myrtaceae	*Myrcia guianensis*
	Rubiaceae	*Guettarda viburnoides*
		Psychotria nuda
	Salicaceae	*Casearia sylvestris*
**Hong Kong**	Altingiaceae	*Liquidambar formosana*
	Lauraceae	*Cinnamomum camphora*
		Litsea cubeba
		Machilus pauhoi
	Malvaceae	*Byttneria aspera*
	Moraceae	*Ficus fistulosa*
		Ficus hispida
	Myrtaceae	*Syzygium operculatum*
	Phyllanthaceae	*Aporusa dioica*
		Bischofia javanica
	Poaceae	*Schizostachyum dumetorum*
**USA (North Carolina)**	Aceraceae	*Acer rubrum*
	Ericaceae	*Rhododendron maximum*
	Fagaceae	*Quercus coccinea*
		Quercus prinus
	Magnoliaceae	*Liriodendron tulipifera*
**Japan**	Aceraceae	*Acer mono*
	Betulaceae	*Carpinus cordata*

		Corylus sieboldiana
	Fagaceae	*Quercus serrata*
	Rosaceae	*Prunus jamasakura*
**Chile**	Monimiaceae	*Peumus boldus*
	Nothofagaceae	*Nothofagus dombeyi*
		Nothofagus obliqua
	Poaceae	*Chusquea quila*
	Verbenaceae	*Rhaphithamnus spinosus*
**Spain**	Apocynaceae	*Nerium oleander*
	Araliaceae	*Hedera helix*
	Betulaceae	*Alnus glutinosa*
	Moraceae	*Ficus carica*
	Oleaceae	*Fraxinus angustifolia*
**USA (Maryland)**	Fagaceae	*Fagus grandifolia*
		Quercus prinus
	Hamamelidaceae	*Hamamelis virginiana*
	Magnoliaceae	*Liriodendron tulipifera*
	Oleaceae	*Fraxinus pennsylvanica*
**Portugal**	Betulaceae	*Alnus glutinosa*
	Fagaceae	*Castanea sativa*
		Quercus robur
	Salicaceae	*Populus nigra*
		Salix atrocinerea
**Argentina**	Grossulariaceae	*Ribes magellanicum*
	Nothofagaceae	*Nothofagus dombeyi*
		Nothofagus pumilio
	Poaceae	*Chusquea culeou*
	Proteaceae	*Embohtrium coccineum*
**USA (New York)**	Aceraceae	*Acer saccharum*
	Fagaceae	*Fagus grandifolia*
		Quercus rubra
	Juglandaceae	*Carya ovata*
	Malvaceae	*Tilia americana*
**Australia (Tasmania)**	Myrtaceae	*Eucalyptus globulus*
		Eucalyptus obliqua
	Rhamnaceae	*Pomaderris apetala*
**France**	Betulaceae	*Alnus glutinosa*
		Corylus avellana
	Fagaceae	*Castanea sativa*
		Fagus sylvatica
		Quercus robur
**Canada**	Aceraceae	*Acer saccharinum*
	Betulaceae	*Betula alleghaniensis*
	Cornaceae	*Cornus amomum*
	Fagaceae	*Fagus grandifolia*
	Malvaceae	*Tilia americana*
	Salicaceae	*Populus grandidentata*
		Salix fragilis
**Poland**	Aceraceae	*Acer pseudoplatanus*
	Betulaceae	*Alnus glutinosa*
		Carpinus betulus
	Fagaceae	*Fagus sylvatica*
**Germany**	Aceraceae	*Acer pseudoplatanus*
	Adoxaceae	*Sambucus nigra*
	Betulaceae	*Alnus glutinosa*
	Fagaceae	*Fagus sylvatica*
	Oleaceae	*Fraxinus excelsior*
**Sweden**	Betulaceae	*Alnus incana*
		Betula pubescens
	Salicaceae	*Salix cinerea* hybrid

Table S2. Locations of streams where litter was collected, and decimal latitude (Lat) and longitude (Lon), altitude (Alt; m asl), percent canopy cover, riparian species richness, number of species collected, and soil class at each site [FAO (2006) World reference base for soil resources 2006. A framework for international classification, correlation and communication. World Soil Resources Reports, 103, Rome, Italy]. As some species were collected at more than one site, the total number of species collected was 151.

Site location	Stream	Lat	Lon	Alt	Canopy	Richness	#Spp	Soil class
Kenya	Kabwe	0.41	37.20	1763	92	20-39	10	Haplic Ferralsols
Ecuador	San Isidro	-0.58	-77.87	2141	94	>40	12	Aluandic Andosols
Colombia	Meléndez	3.22	-76.37	1562	75	>40	6	Haplic Ferralsols
Malaysia	Kroh	3.24	101.64	97	80	>40	11	Haplic Acrisols
Costa Rica	Surá	10.26	-84.00	65	50	<10	10	Haplic Acrisols
Brazil (Brasilia)	Capetinga	-15.96	-47.94	1100	80	>40	10	Haplic Ferralsols
Puerto Rico	Prieta	18.32	-65.82	350	85	>40	11	Haplic Acrisols
Australia (Queensland)	Birthday	-18.98	146.17	795	70	>40	10	Haplic Acrisols
Brazil (Belo Horizonte)	Garcia	-20.21	-43.41	1060	70	>40	10	Haplic Ferralsols
Hong Kong	Tai Po Kau	22.43	114.18	200	75	20-39	11	Haplic Acrisols
USA (North Carolina)	Otto	35.05	-83.42	840	80	<10	5	Haplic Cambisols
Japan	Komori	35.83	138.52	1040	75	10-19	5	Haplic Cambisols
Chile	Nonguén	-36.88	-72.99	130	90	10-19	5	Haplic Acrisols
Spain	Rivera de Huelva	37.95	-6.63	430	80	<10	5	Haplic Luvisols
USA (Maryland)	Patapsco	39.22	-76.73	97	99	<10	5	Haplic Acrisols
Portugal	Cerdeira	40.09	-8.20	543	100	<10	5	Haplic Cambisols
Argentina	Huinquita	-41.36	-71.49	925	90	10-19	5	Aluandic Andosols
USA (New York)	Cascadilla	42.43	-76.45	288	90	10-19	5	Haplic Cambisols
Australia (Tasmania)	Guy Fawkes	-42.90	147.30	250	90	<10	3	Haplic Cambisols
France	Orival	43.44	2.09	473	80	8	5	Haplic Cambisols
Canada	Little Rouge	43.84	-79.19	176	70	10-19	7	Haplic Cambisols
Poland	Sanka	50.06	19.71	246	95	<10	4	Haplic Cambisols
Germany	Lonau	51.70	10.38	510	80	<10	5	Haplic Cambisols
Sweden	Krycklan	64.16	19.50	200	60	<10	3	Haplic Podzols

Fig. S1. Cleveland dotplots showing the distribution of data for each litter trait [nitrogen (N) and phosphorus (P) concentrations, log-transformed N:P ratio, magnesium (Mg) and tannin (Tan) concentrations, and log-transformed specific leaf area (SLA)]. Outliers are denoted by open circles.

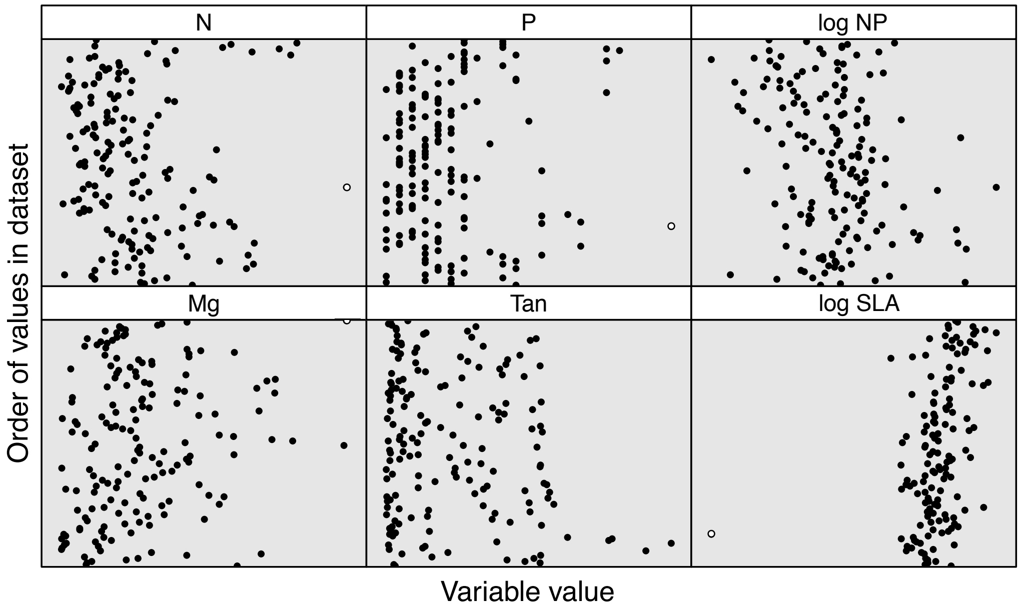

Table S3. Variance inflation factors (VIFs) of litter traits [nitrogen (N) and phosphorus (P) concentrations, log-transformed N:P ratio, magnesium (Mg) and tannin (Tan) concentrations, and log-transformed specific leaf area (SLA)] and environmental variables (mean annual temperature, MAT; mean annual precipitation, MAP; soil pH, SoilpH; and soil N concentration, SoilN) used as predictors in general linear models.

**Predictor**	**VIF**
N	2.718
P	3.035
N x P	2.003
Mg	1.196
Tan	1.367
SLA	1.576
MAT	3.272
MAP	2.279
SoilpH	1.568
SoilN	1.807

Table S4. Summary of backward model selection, based on the Akaike Information Criterion (AIC), for each litter trait. The p-value (*p*) refers to the comparison between 1st and 2nd, 2nd and 3rd model, and so on; non-significant values ($p > 0.05$) indicate that both models are similar. MAT, mean annual temperature; MAP, mean annual precipitation; soilpH, soil pH; soilN, soil nitrogen concentration.

Model	df	AIC	*p*
**Nitrogen concentration (N)**			
N ~ P + Mg + Tan + MAT + MAP + MAT×MAP + soilpH + soilN	33	227.4	
N ~ P + Tan + MAT + MAP + MAT×MAP + soilpH + soilN	32	225.4	0.87
N ~ P + Tan + MAT + MAP + MAT×MAP + soilpH	31	224	0.43
**Phosphorus concentration (P)**			
P ~ N + Mg + Tan + MAT + MAP + MAT×MAP soilpH + soilN	33	-699.5	
P ~ N + Mg + Tan + MAT + MAP + soilpH + soilN	32	-701.5	0.82
P ~ N + Tan + MAT + MAP + soilpH + soilN	31	-703	0.52
P ~ N + Tan + MAT + soilpH + soilN	30	-704.7	0.57
P ~ N + Tan + MAT + soilpH + soilN	29	-705.2	0.22
**N:P ratio (NP)**			
log(NP) ~ Mg + Tan + MAT + MAP + MAT×MAP + soilpH + soilN	32	298.2	
log(NP) ~ Tan + MAT + MAP + MAT×MAP+ soilpH + soilN	31	296.2	0.77
log(NP) ~ Tan + MAT + MAP + MAT×MAP + soilpH	30	294.4	0.74
log(NP) ~ Tan + MAT + MAP + MAT×MAP	29	294.1	0.19
**Magnesium concentration (Mg)**			
Mg ~ N + P + NP + Tan + MAT + MAP + MAT×MAP + soilpH + soilN	34	836	
Mg ~ N + P + NP + MAT + MAP + MAT×MAP + soilpH + soilN	33	834	0.99
Mg ~ N + P + NP + MAT + MAP + soilpH + soilN	32	834.5	0.11
Mg ~ N + P + NP + MAP + soilpH + soilN	31	832.5	0.94
**Condensed tannin concentration (Tan)**			
Tan ~ N + P + NP + Mg + MAT + MAP + MAT×MAP + soilpH	34	1169.7	
Tan ~ N + P + NP + Mg + MAT + MAP + MAT×MAP + soilN + soilpH	33	1169.1	0.23
Tan ~ N + NP + Mg + MAT + MAP + MAT×MAP + soilpH	32	1168.7	0.21
Tan ~ N + Mg + MAT + MAP + MAT×MAP + soilpH	31	1166.8	0.67

**Specific leaf area (SLA)**			
log(SLA) ~ N + P + NP + Mg + Tan + MAT + MAP + MAT×MAP + soilN + soilpH	34	150.6	
log(SLA) ~ N + P + NP + Mg + Tan + MAT + MAP + MAT×MAP + soilN	33	148.9	0.56
log(SLA) ~ N + P + Mg + Tan + MAT + MAP + MAT×MAP + soilN	32	147.6	0.41
log(SLA) ~ N + P + Tan + MAT + MAP + MAT×MAP + soilN	31	146.9	0.26
log(SLA) ~ N + P + Tan + MAT + MAP + soilN	30	146.3	0.23
log(SLA) ~ N + P + Tan + MAT + soilN	29	144.3	0.96
log(SLA) ~ P + Tan + MAT + soilN	28	144	0.19

Supplementary Methods. Phyton 2.7. tool ‘leafbud.py’

```
import ete3
from ete3 import Tree
from ete3 import NCBITaxa
ncbi = NCBITaxa()

def recursive_place(node,lineage) x  #places nodes based on lineage
    placedict = {}
    for child in node.get_children() x
        cName = " ".join(child.get_leaves()[0].name.split("_"))
        cLin = ncbi.get_lineage(ncbi.get_name_translator([cName])[cName][0])
        cLin = cLin[0 x len(lineage)]
        placedict.setdefault(child,cLin)
    i=0
    for key in placedict.keys() x
        x = set(placedict[key]).intersection(set(lineage))
        x=len(x)
        if x == i x
            i=x
            choice = node
        if x > i x
            i = x
            choice = key
    if i == 0 x
        print "MRCA",node
        print "LINEAGE",lineage
        print node.get_children()
        print node.dist
        choice = node
    return(choice)

aBplaced = "needed_tips_3.txt" #file with names of tips to be placed, separated by newlines
tree2 = "R_pruned_tree_round2" #tree to place tips on in newick notation
outtree = "output.tree" #name of the output file
outgroup =
"Pollia_macrophylla,Gigantochloa_scortechinii,Chusquea_culeou,Prestoea_acuminata,Chusquea_quila
"#outgroup

still_missing = []
```

```
tree = Tree(tree2)
with open(aBplaced,'r') as place x
    x = place.read().split("\n")
    missing = list(set(x)-set(tree.get_tree_root().get_leaf_names()))
    print "Number to be added x  ", len(missing)
    for line in missing x
        geList = []
        if len(line) < 4 x
            continue
        print "placing ",line
        genus = line.split("_")[0]
        species = line.split("_")[1]
        for taxa in tree.iter_leaves() x
            if taxa.name.split("_")[0] == genus x
                geList.append(taxa)
        if len(geList) == 0 x
            print "Genus not found, searching family"
            still_missing.append(line)
            ncbID = ncbi.get_name_translator([genus])[genus]
            lineage = ncbi.get_lineage(ncbID[0])
            ranks = ncbi.get_rank(lineage)
            for rankID in ranks.keys() x
                if ranks[rankID] == u'family' x
                    familyID = ncbi.get_taxid_translator([rankID])[rankID]
                    for node in tree.traverse(strategy="postorder") x
                        if node.name == str(familyID) x
                            placedict = {}
                            rnode = recursive_place(node,lineage)
                            while rnode != node x
                                node = rnode
                                rnode = recursive_place(node,lineage)
                            print "Family found & placed"
                            mrca = rnode
                            if len(mrca.get_children()) == 0 x
                                print "Closest Family was OTU, placing as sister"
                                blen = mrca.dist
                                mrca.up.add_child(name=line,dist=blen)
                            else x
                                blen = mrca.get_distance(mrca.get_leaves()[0])
                                mrca.add_child(name=line,dist=blen)
                            still_missing.remove(line)
                    if line in still_missing x
                        x = 0
                        i = 0
                        choice = 0
                        for node in tree.iter_leaves() x
                            nodeID = ncbi.get_name_translator([node.name.split("_")[0]])
                            nodeLin = ncbi.get_lineage(nodeID[node.name.split("_")[0]][0])
                            x = set(nodeLin).intersection(set(lineage))
                            x=len(x)
                            if x > i x
                                i = x
                                choice = node
                        if i !=0 x
                            print "Discovered closeset relative as OTU, placing as sister."
                            blen = choice.dist
                            choice.up.add_child(name=line,dist=blen)
            if line == "Emmotum_nitens" x  #certain genera are problematic, manually assign their location
                mrca = tree.get_common_ancestor("Fraxinus_profunda","Plocosperma_buxifolium")
                blen = mrca.get_distance("Fraxinus_profunda")
```

```
            mrca.add_child(name = line, dist = blen)
            print "added Emmotum"
        else x
          if len(geList)==1 x
            print "Only one species in genus, placing as sister"
            mrca = geList[0].up
          else x
            print "Genus found, placing at MRCA"
            mrca = tree.get_common_ancestor(geList)
          blen = mrca.get_distance(geList[0])#split branch lengths on new node
          mrca.add_child(name=line,dist=blen)
tree.write(outfile = outtree,format=1)
aBplaced = "missing_taxa_iter2"
with open(aBplaced,'w') as iter2 x
    for i in still_missing x
        iter2.write("{0}\n".format(i))

outgroup = tree.get_common_ancestor(outgroup.split(","))
#print "Pruning, this may take several minutes..."
#tree.prune(x,preserve_branch_length=True)

tree.write(outfile = "Pruned_tree", format = 1)
print"pruned and saved..randomly resolving polytomies because R can't handle itself"
tree.resolve_polytomy(recursive=True)
#print"rooting at user defined outgroup because R is a little baby"
#tree.set_outgroup(outgroup)
for zeroNode in tree.search_nodes(dist=0) x
    zeroNodeChildren = zeroNode.get_children()
    if zeroNode.is_leaf() == True x
        print "LEAF",zeroNode
    if len(zeroNodeChildren) > 1 and zeroNode.is_root() == False x
        smallNode = min([zero.dist for zero in zeroNodeChildren])/2
        print "Min node dist x  ",smallNode
        for node in zeroNodeChildren x
            node.dist = node.dist - smallNode
        zeroNode.dist = smallNode

tree.write(outfile = "%s_tree2" % outtree)
```
